# Glycemic Control and Mortality in Diabetic Patients Undergoing Dialysis Focusing on the Effects of Age and Dialysis Type: A Prospective Cohort Study in Korea

**DOI:** 10.1371/journal.pone.0136085

**Published:** 2015-08-18

**Authors:** Ji In Park, Eunjin Bae, Yong-Lim Kim, Shin-Wook Kang, Chul Woo Yang, Nam-Ho Kim, Jung Pyo Lee, Dong Ki Kim, Kwon Wook Joo, Yon Su Kim, Hajeong Lee

**Affiliations:** 1 Department of Internal Medicine, Kangwon National University College of Medicine, Chuncheon, Korea; 2 Clinical Research Center of End Stage Renal Disease in Korea, Daegu, Korea; 3 Department of Internal Medicine, Seoul National University College of Medicine, Seoul, Korea; 4 Department of Internal Medicine, Kyungpook National University School of Medicine, Daegu, Korea; 5 Department of Internal Medicine, Yonsei University College of Medicine, Seoul, Korea; 6 Department of Internal Medicine, The Catholic University of Korea College of Medicine, Seoul, Korea; 7 Department of Internal Medicine, Chonnam National University Medical School, Gwangju, Korea; 8 Department of Internal Medicine, Seoul National University Boramae Medical Center, Seoul, Korea; Massachusetts General Hospital, UNITED STATES

## Abstract

**Background:**

Active glycemic control has been proven to delay the onset and slow the progression of diabetic retinopathy, nephropathy, and neuropathy in diabetic patients, but the optimal level is obscure in end-stage renal disease. In this study, we evaluated the effect of hemoglobin A1c (HbA1c) on mortality of diabetic patients on dialysis, focusing on age and dialysis type.

**Methods:**

Of 3,302 patients enrolled in the prospective cohort for end-stage renal disease in Korea between August 2008 and October 2013, 1,239 diabetic patients who had been diagnosed with diabetes or having HbA1c≥6.5% at the time of enrollment were analyzed. Age was categorized as <55, 55–64 and ≥65 years old. Age, sex, modified Charlson comorbidity index, hemoglobin, primary renal disease, body mass index, and dialysis duration were adjusted.

**Results:**

A total of 873 patients received hemodialysis (HD) and 366 underwent peritoneal dialysis (PD). During the mean follow-up of 19.1 months, 141 patients died. Patients with poor glucose control (HbA1c≥8%) showed worse survival than patients with HbA1c<8% (hazard ratio [HR], 2.2; 95% confidence interval [CI], 1.48–3.29; *P*<0.001). Subgroup analysis divided by age revealed that HbA1c≥8% was a predictor of mortality in age <55 (HR, 4.3; 95% CI, 1.78–10.41; *P* = 0.001) and age 55–64 groups (HR, 3.3; 95% CI, 1.56–7.05; *P* = 0.002), but not in age ≥65 group. Combining dialysis type and age, poor glucose control negatively affected survival only in age < 55 group among HD patients, but it was significant in age < 55 and age 55–64 groups in PD patients. Deaths from infection were more prevalent in the PD group, and poor glucose control tended to correlate with more deaths from infection in PD patients (*P* = 0.050).

**Conclusions:**

In this study, the effect of glycemic control differed according to age and dialysis type in diabetic patients. Thus, the target of glycemic control should be customized; further observational studies may strengthen the clinical relevance.

## Introduction

Strict glycemic control has been proven to delay the onset and slow the progression of diabetic retinopathy, nephropathy, and neuropathy in patients with diabetes mellitus (DM) [1, 2]. Based on cumulative evidence, the American Diabetes Association (ADA) recommends reasonable hemoglobin A1c (HbA1c) goal for many nonpregnant adults as <7.0% [3].

Many diabetic patients develop diabetic nephropathy during the long disease course. Currently, DM is the most common etiology of end stage renal disease (ESRD) in many countries [4, 5]. However, the evidence regarding glycemic control targets for those DM patients on dialysis has been very scarce. The representative clinical practice guidelines for kidney disease published several years ago, the Kidney Disease Outcomes Quality Initiative (KDOQI) and Kidney Disease Improving Global Outcomes (KDIQO) guidelines, recommended levels of HbA1c<7% for chronic kidney disease or ESRD patients on the basis of weak evidence from clinical trials that excluded ESRD patients [6, 7].

In recent years, this field has become a focus of interest. Noteworthy is a meta-analysis investigating 10 studies that revealed levels of HbA1c≥8.5% were associated with higher mortality in diabetic patients receiving hemodialysis (HD) [8]. For patients with peritoneal dialysis (PD), poor glycemic control with HbA1c≥8% appeared to have an adverse effect adversely on survival in a large-scale study [9]. Interestingly, one study reported the glycemic control is more important in younger patients below 60 years of age [10].

We aimed to investigate the association between HbA1c and mortality in a large Asian cohort including both HD and PD patients. To suggest an individualized target for glycemic control, we focused on the effects of dialysis modality and age.

## Materials and Methods

### Study design and population

This study was part of prospective cohort study of the Clinical Research Center for End Stage Renal Disease (CRC for ESRD) in South Korea. It is a nationwide web-based multi-center prospective cohort study of patients with ESRD, designed to improve survival rate and quality of life and to create effective treatment guidelines (clinicaltrial.gov NCT00931970). Thirty-one hospitals and clinics in Korea participated, and patients aged 18 years or more with ESRD who were initiated on dialysis were enrolled. Over a 5-year period (August 2008 through October 2013), a total of 3,302 patients were enrolled in CRC for ESRD. All patients provided their written consent to participate in this study, which was approved by the institutional review board at each participating center (please see S1 Text for full names). All clinical investigations were conducted in accordance with the guidelines of the 2008 Declaration of Helsinki.

From this cohort, we analyzed patients who had been diagnosed with DM or having HbA1c≥6.5% at the time of enrollment. Overall, 1,542 (46.7%) patients had DM and 65 (2.0%), who had not been diagnosed DM, showed HbA1c≥6.5%. After excluding 368 diabetic patients with no available HbA1c data, 1,239 patients were evaluated in this study.

### Measurements

In CRC for ESRD, both clinical and laboratory data had been stored in the form of web-based medical questionnaires. The questionnaire items were filled in by data coordinators, who were trained in each center to collect patient data through a combination of chart reviews and direct interviews using a standardized form. The HbA1c level was verified and recorded as outlined above at cohort enrollment and after every 12 months. For survival analyses, the HbA1c level was categorized into three groups defined as <6.5%, 6.5–7.9%, and ≥8%. Age at enrollment was also categorized into three groups according to age as follows: <55, 55–64, and ≥65 years old. Next, we analyzed focusing on the change of HbA1c from baseline to 1 year follow-up. Because there were missing values, a total of 574 patients were analyzed. We divided them into four groups according to their HbA1c change; <8% to <8%, <8% to ≥8%, ≥8% to <8%, and ≥8% to ≥8%.

The modified Charlson co-morbidity index (MCCI) was calculated for each patient. MCCI was developed to predict one-year mortality, and it has been validated in ESRD patients [11, 12]. MCCI score is composed of 22 comorbidities including myocardial infarction, congestive heart failure, peripheral vascular disease, cerebrovascular disease, dementia, chronic pulmonary disease, connective tissue disease, ulcer disease, mild liver disease, diabetes, hemiplegia, moderate or severe renal disease, diabetes with end organ damage, any tumor, leukemia, lymphoma, moderate or severe liver disease, metastatic solid tumor, and acquired immune deficiency syndrome.

In addition, the following demographic and clinical data were collected and analyzed in this study: sex, dialysis duration, primary renal disease, type of DM, body mass index (BMI), antihypertensive medications, laboratory values including hemoglobin, albumin, cholesterol, and high-sensitivity CRP (hsCRP), and comorbidities including coronary artery disease, peripheral vascular disease, cerebrovascular disease, congestive heart failure, and malignancy.

### Outcomes

The primary outcome was all-cause mortality and the secondary outcome was mortality due to cardiovascular disease and infection. Each center recorded information regarding mortality and cause of death on the CRC for ESRD web-based registry. Research coordinators from a centralized center carried out a regular sample survey on about 20 percent of the enrolled patients to confirm the medical records twice a year. All the medical records of patients who died in hospital registered in CRC for ESRD were checked to confirm the cause-specific death and the mortality date. In the case of patient death in other hospitals, information of cause-specific death was extracted from the Korean National Statistical Office data as of December 31, 2011.

### Statistical Analyses

Analyses of the differences in the baseline characteristics between HD and PD were performed using the *t* test for continuous variables and the chi-square test for categorical variables. The Kaplan-Meier method was used to compare survival curves, and differences were assessed by means of the log rank test. The multivariate Cox proportional hazards regression models were used to examine the association between HbA1c levels and survival in both unadjusted and adjusted models. We adjusted for risk factors for death that were also plausibly related to blood glucose levels. Potential confounders included age, sex, MCCI, primary renal disease, BMI, and dialysis duration. In addition, we adjusted for hemoglobin levels because anemia can affect the interpretation of HbA1c levels. The assumption of linearity for the Cox models was examined through visual inspection, and no violation of proportional hazards was found. The effects of the multivariate Cox proportional hazards regression models are shown as hazard ratio (HR) and 95% confidence index (CI).

Statistical analysis was performed using SPSS version 21.0 (SPSS, Inc., Chicago, Illinois, USA). For all analysis, results were considered statistically significant if *P*<0.050.

## Results

### Patient characteristics according to dialysis modality

A total of 1,239 patients were analyzed during the mean follow-up of 19.1 months, during this period 141 patients (11.4%) died. The baseline patient characteristics are summarized according to dialysis modality in Table 1. Of the 1,239 patients, 873 and 366 patients were on HD and PD, respectively. The mean age was 59.4±11.6 years old and PD patients were significantly younger than HD patients. There are more prevalent patients in HD group than those in PD group and the mean dialysis duration of prevalent patients are 3.8±4.1 years. In both groups, DM was the most dominant cause of ESRD. Among PD patients, the proportion of non-diabetic ESRD was significantly higher. The MCCI score was higher in the HD group, and indicates more severe comorbid status. Regarding antihypertensive medication, renin-angiotensin system inhibitors were used more among PD patients. The mean HbA1c level was 6.9±1.4 and was not significantly different between the two groups, while the HD group showed lower hemoglobin, higher albumin, and lower cholesterol levels than those in the PD group.

**Table 1:** Patient characteristics according to dialysis type.

Characteristics	Total (N = 1,239)	HD (N = 873)	PD (N = 366)	*P*
**Age (year)**	59.4 ± 11.6	60.6 ± 11.5	56.4 ± 11.4	<0.001
**Sex (male, %)**	61.4	60.7	63.1	0.428
**Patient classification (%)**				
Incident	55.9	58.8	48.9	0.001
Prevalent	44.1	41.2	51.1	
**Dialysis duration (prevalent only, year)**	3.8 ± 4.1	3.9 ± 4.3	3.3 ± 2.5	0.032
**Cause of ESRD (%)**				
Diabetes mellitus	88.9	90.4	85.2	0.007
Other than diabetes mellitus	10.8	9.3	14.5	
Unknown	0.3	0.3	0.3	
**Type of diabetes mellitus (%)**				
Type 1	3.6	3.3	4.4	0.272
Type 2	81.5	83.3	77.3	
Unknown	14.9	13.4	18.3	
**Blood pressure (mmHg)**				
Systolic blood pressure	142.9 ± 22.7	145.1 ± 22.5	137.4 ± 22.4	<0.001
Diastolic blood pressure	76.4 ± 13.2	75.8 ± 13.3	77.7 ± 12.9	0.028
**BMI (kg/m** ^**2**^ **)**	23.3 ± 3.4	23.2 ± 3.4	23.7 ± 3.3	0.013
**MCCI**	6.3 ± 2.2	6.5 ± 2.2	5.9 ± 2.1	<0.001
**Comorbidities (%)**				
Coronary artery disease	20.5	20.6	20.2	0.893
Peripheral vascular disease	12.1	10.6	15.8	0.011
Cerebrovascular disease	11.1	10.9	11.6	0.718
Congestive heart failure	14.3	13.9	15.4	0.473
Malignancy	5.7	7.2	2.2	0.001
**Medications (%)**				
RAS blockers	54.6	52.5	59.8	0.018
Calcium channel blockers	57.1	55.6	60.7	0.102
ß-blockers	55.8	54.8	58.2	0.287
Diuretics	58.9	59.5	57.7	0.569
**Laboratory values**				
Hemoglobin A1c (%)	6.9 ± 1.4	6.9 ± 1.4	7.0 ± 1.5	0.166
Hemoglobin (g/dL)	9.7 ± 1.7	9.5 ± 1.7	10.0 ± 1.6	<0.001
Albumin (g/dL)	3.5 ± 0.6	3.5 ± 0.6	3.4 ± 0.6	0.047
Total cholesterol (mg/dL)	157.8 ± 48.4	152.6 ± 46.7	170.0 ± 50.0	<0.001
hsCRP (mg/dL)	2.8 ± 12.6	3.0 ± 13.6	2.1 ± 9.6	0.256

HD, hemodialysis; PD, peritoneal dialysis; ESRD, end-stage renal disease; BMI, Body mass index; MCCI, Modified Charlson co-morbidity index; RAS; renin-angiotensin system

### Patient characteristics by age

Clinical characteristics were significantly different according to the three age groups (Table 2). The mean ages for age <55, 55–64, and ≥65 groups were 46.2, 50.4, and 71.3 years, respectively. In age ≥65 group, the proportion of prevalent dialysis seemed to be higher and dialysis duration longer, but they were not significantly different. In age <55 group, type 1 DM was more prevalent than in older patients. The MCCI score showed a higher value with aging. Several comorbidities including coronary artery disease, cerebrovascular disease, and malignancy were more common in age ≥65 group. Among laboratory tests, the HbA1c level was not significantly different among the three age groups.

**Table 2:** Patient characteristics according to age.

Characteristics	Age < 55 (N = 407)	Age 55–64 (N = 379)	Age ≥ 65 (N = 453)	*P*
**Sex (male, %)**	60.4	62.5	61.4	0.834
**Patient classification (%)**				
Incident	59.0	54.9	53.9	0.290
Prevalent	41.0	45.1	46.1	
**Dialysis duration (prevalent only, year)**	3.1 ± 2.8	3.8 ± 4.2	4.1 ± 4.1	0.054
**Cause of ESRD (%)**				
Diabetes mellitus	88.7	91.0	87.2	0.519
Other than diabetes mellitus	11.1	8.7	12.4	
Unknown	0.2	0.3	0.4	
**Type of diabetes mellitus (%)**				
Type 1	8.6	1.8	0.7	<0.001
Type 2	76.9	86	81.9	
Unknown	14.5	12.1	17.4	
**Blood pressure (mmHg)**				
Systolic blood pressure	145.2 ± 23.4	141.5 ± 23.3	142.0 ± 21.4	0.046
Diastolic blood pressure	80.8 ± 12.9	75.6 ± 13.0	73.0 ± 12.6	<0.001
**BMI (kg/m** ^**2**^ **)**	23.3 ± 3.5	23.6 ± 3.1	23.2 ± 3.5	0.170
**MCCI**	5.0 ± 1.8	6.4 ± 1.9	7.5 ± 2.0	<0.001
**Comorbidities (%)**				
Coronary artery disease	9.2	23.5	28.2	<0.001
Peripheral vascular disease	11.2	13.2	23.1	0.694
Cerebrovascular disease	5.9	9.9	16.7	<0.001
Congestive heart failure	11.9	13.9	17.0	0.100
Malignancy	3.2	6.2	7.6	0.020
**Medications (%)**				
RAS blockers	54.3	55.4	54.3	0.937
Calcium channel blockers	58.0	60.2	58.7	0.821
ß-blockers	60.0	54.1	53.4	0.115
Diuretics	58.5	56.5	56.3	0.780
**Laboratory values**				
HbA1c (%)	6.9 ± 1.6	7.0 ± 1.4	6.8 ± 1.3	0.247
Hemoglobin (g/dL)	9.5 ± 1.8	9.7 ± 1.6	9.8 ± 1.6	0.015
Albumin (g/dL)	3.4 ± 0.7	3.5 ± 0.6	3.5 ± 0.6	0.004
Total cholesterol (mg/dL)	166.5 ± 55.6	157.9 ± 45.6	149.7 ± 41.7	<0.001
hsCRP (mg/dL)	1.7 ± 7.9	2.1 ± 8.9	4.2 ± 17.3	0.012

HD, hemodialysis; PD, peritoneal dialysis; ESRD, end-stage renal disease; BMI, Body mass index; MCCI, Modified Charlson co-morbidity index; RAS; renin-angiotensin system

### Survival according to HbA1c levels: the effect of age and dialysis modality

**Fig 1:**
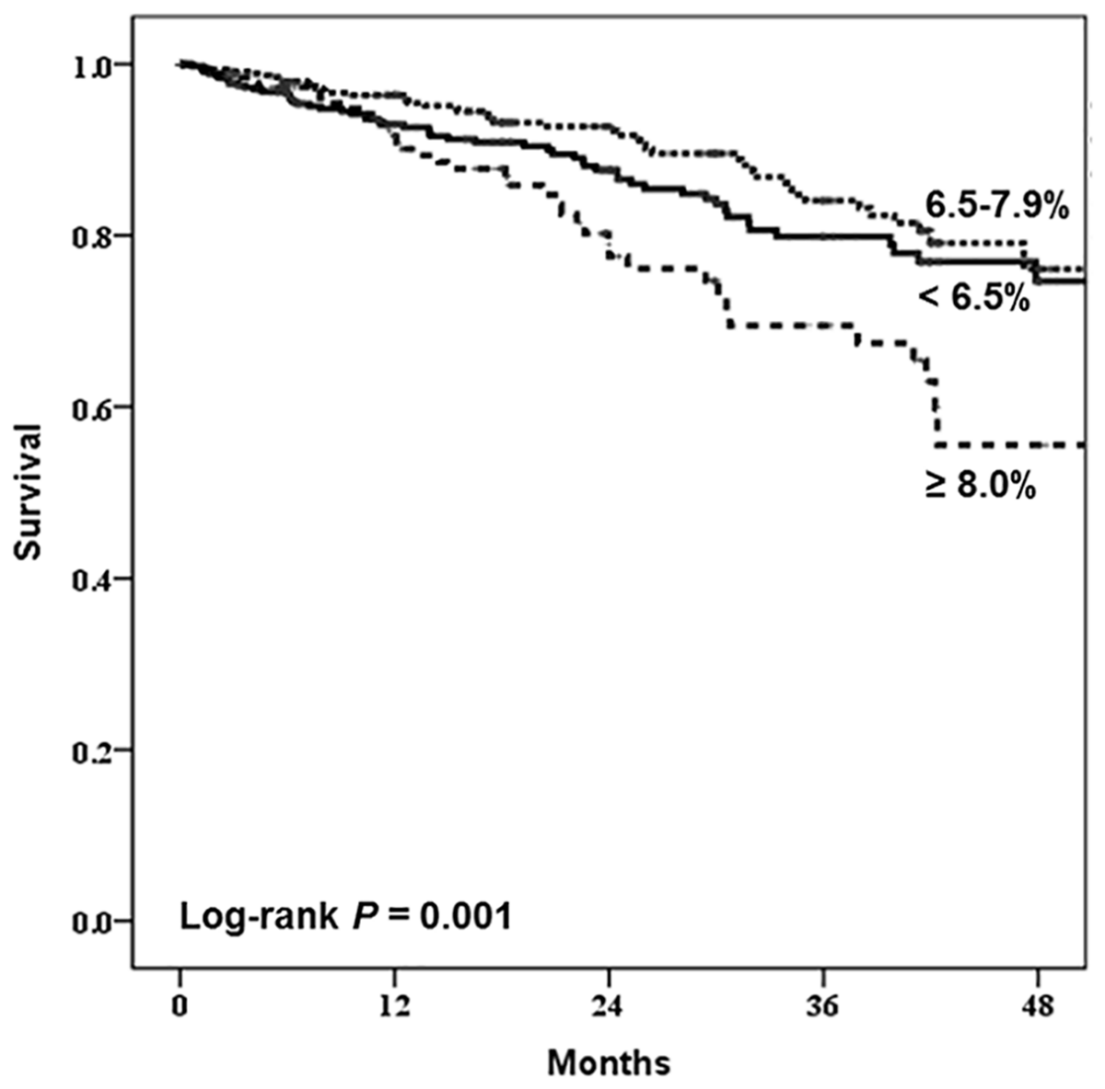
Kaplan-Meier survival curve for all-cause mortality according to HbA1c levels (%).

**Fig 2:**
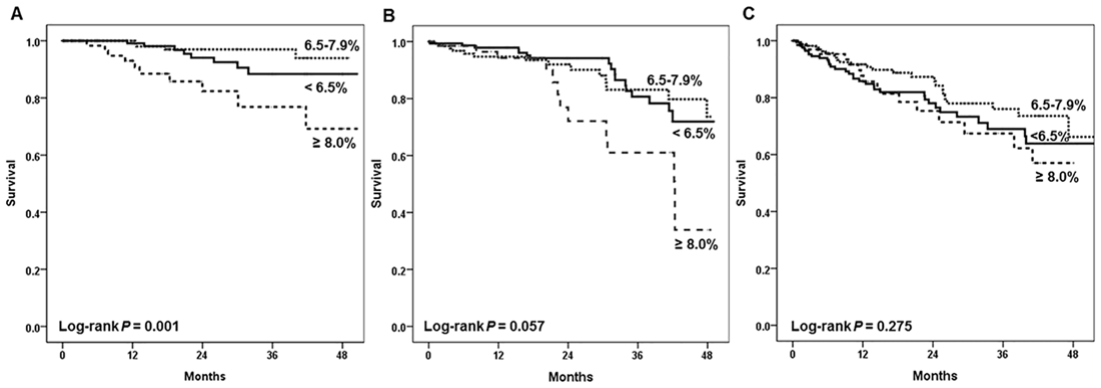
Kaplan-Meier survival curves for all-cause mortality by HbA1c (%). (A) Age <55 years old (B) Age 55–64 years old, and (C) Age ≥65 years old.

**Fig 3:**
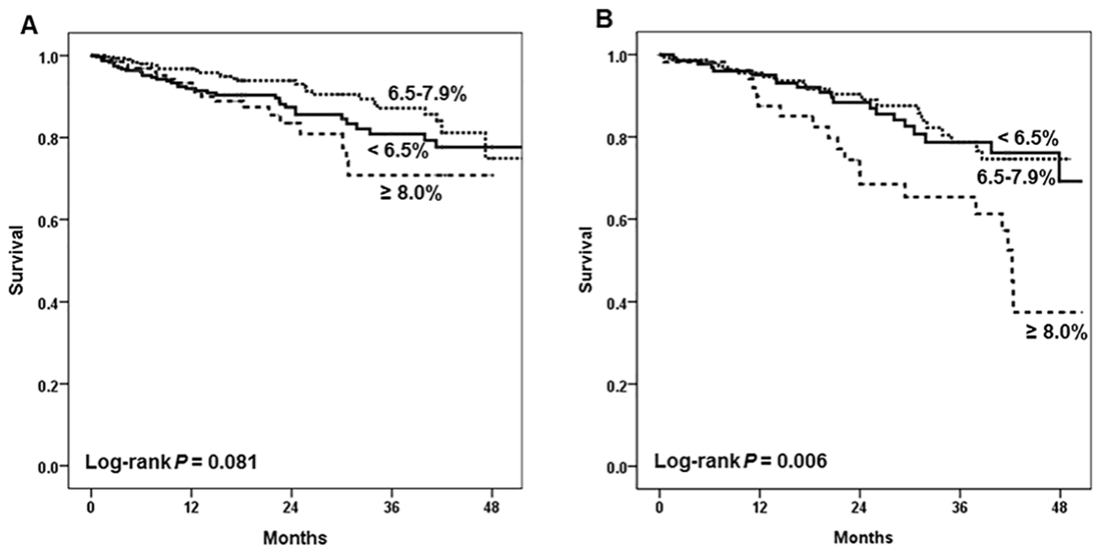
Kaplan-Meier survival curves for all-cause mortality by HbA1c (%). (A) Hemodialysis patients and (B) Peritoneal dialysis patients.

In the Kaplan-Meier analysis, patients with HbA1c≥8% showed significantly lower survival among the 1,239 patients examined (Fig 1). When analyzed according to the subgroups divided by age, the HbA1c level apparently influenced the survival of the group aged below 55 years old, but the effect was attenuated by aging (Fig 2). We also analyzed similar effects in the HD and PD group respectively (Fig 3). In the HD group, glycemic control tended to affect overall survival but it was not statistically significant. On the other hand, it is worth noting that the HbA1c≥8% group showed significantly worse survival among PD patients (*P* = 0.006). When we compared the mortality according to the subgroups divided by dialysis duration, HbA1c≥8% group showed worse survival among patients with dialysis duration less than 3 years (S1 Fig). Next, we analyzed mortality focusing on the change of HbA1c, baseline HbA1c level seemed to more significantly affect patient survival than 1 year follow-up value did, especially among incident dialysis patients (S2 Fig).

Next, we performed univariate and multivariate Cox analysis. Because there were no significant differences in survival between the HbA1c<6.5% and 6.5–7.9% group in the aforementioned analyses, the HbA1c level was re-categorized simply as below and above 8%. As shown in Table 3, the HbA1c≥8% group revealed worse survival than the HbA1c<8% group (adjusted HR, 2.20; 95% CI, 1.48–3.29; *P*<0.001). In the subgroup analysis, similar results were shown in the HD patients (adjusted HR, 1.86; 95% CI, 1.08–3.21; *P* = 0.025) and the PD group (adjusted HR, 2.68; 95% CI, 1.38–5.20; *P* = 0.004). However, when analyzed in the three groups subdivided by age, HbA1c≥8% was a significant predictor in age <55 (adjusted HR, 4.30; 95% CI, 1.78–10.41; *P* = 0.001) and in age 55–64 groups (adjusted HR, 3.32; 95% CI, 1.56–7.05; *P* = 0.002), but not in age ≥65 group.

**Table 3:** Univariate and multivariate Cox analysis.

HbA1c	unadjusted HR (95% CI)	*P*	adjusted HR (95% CI)*	*P* *
**Total (N = 1,239)**				
< 8%	reference		reference	
≥ 8%	1.90 (1.31–2.76)	0.001	2.13 (1.44–3.16)	<0.001
**Hemodialysis (N = 873)**				
< 8%	reference		reference	
≥ 8%	1.90 (1.06–3.43)	0.032	1.84 (1.08–3.14)	0.025
**Peritoneal dialysis (N = 366)**				
< 8%	reference		reference	
≥ 8%	2.49 (1.33–4.66)	0.004	2.23 (1.27–3.93)	0.006
**< 55 years (N = 407)**				
<8%	reference		reference	
≥8%	4.14 (1.79–9.60)	0.001	4.48 (1.90–10.56)	0.001
**55–64 years (N = 379)**				
<8%	reference		reference	
≥8%	2.21 (1.13–4.32)	0.020	3.13 (1.51–6.48)	0.002
**≥ 65 years (N = 453)**				
<8%	reference		reference	
≥8%	1.32 (0.76–2.30)	0.322	–	–

* Adjusted for age, sex, modified Charlson comorbidity index, hemoglobin, primary renal disease, body mass index, and dialysis duration

**Fig 4:**
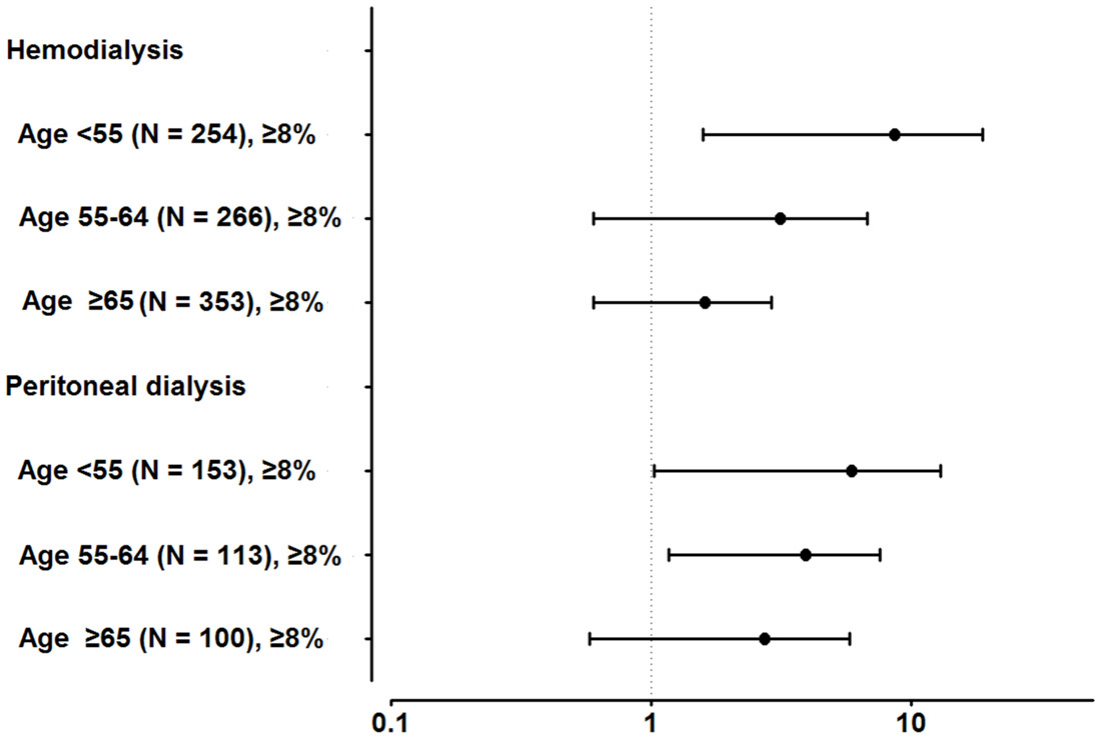
Multivariate analysis in subgroups according to dialysis modality and age Adjusted for age, sex, modified Charlson comorbidity index, hemoglobin, primary renal disease, body mass index, and dialysis duration. Reference groups are patients with HbA1c<8% in each population.

For further subgroup analyses, we divided the population into six groups according to the dialysis modality and age. Among the HD patients, HbA1c≥8% negatively affected survival only in age <55 group (adjusted HR, 5.45; 95% CI, 1.58–18.75; *P* = 0.007) (Fig 4). However, in the PD patients, the effect of HbA1c was significant after multivariate analysis in age <55 (adjusted HR, 3.65; 95% CI, 1.03–12.97; *P* = 0.045) and age 55–64 groups (adjusted HR, 2.98; 95% CI, 1.17–7.58; *P* = 0.022)

### Cause of death according to dialysis modality

In addition, we investigated the cause of death in the population. While cardiovascular disease was the leading cause of death in the HD group (21.69 per 1000-patient-year), infection was the predominant cause of death in the PD group (32.20 per 1000-patient-year) (Table 4).

**Table 4:** Cause of death according to dialysis type.

Cause of death	HD	PD	Total
Cardiovascular disease	21.69	27.81	23.79
Infection	20.14	32.20	24.29
Other	10.85	14.64	12.15
All-cause	52.68	74.65	60.23

per 1000-patient-year

**Fig 5:**
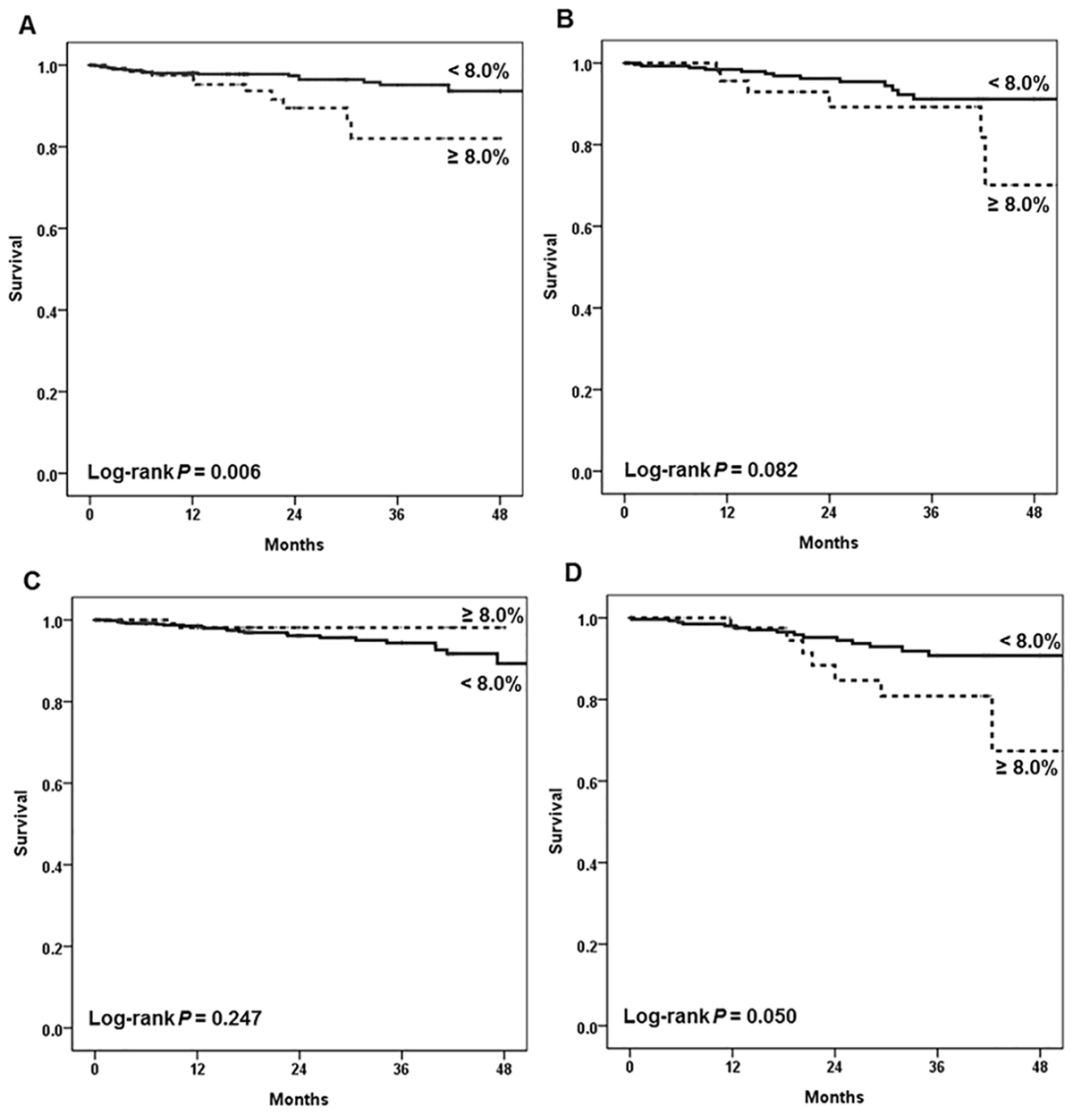
Kaplan-Meier survival curves for cause-specific death according to HbA1c level (A) Death from cardiovascular disease in hemodialysis patients, (B) Death from cardiovascular disease in peritoneal dialysis patients, (C) Death from infection in hemodialysis patients, and (D) Death from infection in peritoneal dialysis patients.

Finally, mortality due to cardiovascular disease or infection respectively was analyzed using the Kaplan-Meier method both in the HD and PD groups. As for cardiovascular death, glycemic control influenced only the hemodialysis group. However, in deaths from infection, patients with HbA1c≥8% showed a worse survival only in the patients who underwent PD (*P* = 0.050, marginally significant) (Fig 5).

## Discussion

We evaluated the effects of glycemic control on the mortality of ESRD patients with DM in a large Asian cohort. Poor glycemic control, defined as HbA1c≥8%, was demonstrated to negatively affect survival in both the HD and PD groups. Subgroup analysis dividing cases by age revealed that glycemic control was not associated with mortality in patients above 65 years old. Interestingly, the effect was not significant in patients above 55 years old undergoing HD, not in those above 65 years old undergoing PD.

The association between HbA1c and mortality in the ESRD population with DM has been studied extensively, especially in patients who undergo HD. Ramirez et al. reported findings from the Dialysis Outcomes and Practice Patterns Study (DOPPS), which revealed a U-shaped association between HbA1c and mortality [13]. Both poor and strict glycemic control appeared to be associated with higher mortality rates. A recent meta-analysis including 83,684 participants across 10 studies concluded that high HbA1c levels (≥8.5%) were associated with high all-cause mortality risk in diabetic patients under HD [8]. There are also several studies demonstrating that HbA1c levels above 8% adversely affect survival [14, 15]. We used the 8% cut-off because in our cohort, the population with HbA1c over 8.5% consisted of only 145 patients (11.7%), which was too small to perform further subgroup analysis. For PD patients with DM, relatively little evidence has been reported and is based on only 5 studies to date [9, 16–19]. In the largest study among the five, Duong et al. reported that time-averaged HbA1c≥8% was associated with a highest risk of all-cause mortality after analyzing 2,798 PD patients with DM [9]. Our results derived from both HD and PD patients are consistent with previous studies.

Of note, in the present study, we conducted our analysis according to age groups, which showed interesting results. The effects of higher HbA1c levels on survival attenuated with aging, and disappeared in the patients aged over 65 years. Since randomized controlled trials have not included many older patients, the benefits of intensive treatment of hyperglycemia in older diabetics are uncertain. There are several retrospective cohort studies evaluating the association between HbA1c levels and morality in older diabetic patients [20, 21]. Based on these results, a consensus report by the ADA and the American Geriatrics Society firstly suggested HbA1c goals for patients above 65 years old as follows: healthy, <7.5%; complex/intermediate, <8.0%; very complex/poor health status, <8.5% [22]. The latest ADA recommendations keep the same opinion [23]. Meanwhile, there is no consensus for older diabetic patients under dialysis. Recently, initial evidence from the national UK Renal Registry data has been published [10]. Adler et al. investigated 3,157 diabetic patients on dialysis, and found that HbA1c levels exceeding 8.5% were associated with poorer survival only in patients less than 60 years of age. Likewise, our data showed that the survival of the oldest group aged over 65 years old was not affected by elevated HbA1c levels.

The follow-up studies of the Diabetes Control and Complications Trial (DCCT) and the United Kingdom Prospective Diabetes Study (UKPDS) showed the long-term benefits of earlier periods of intensive glucose control with regards to macrovascular complications and mortality [24, 25]. However, data from the recently published Action in Diabetes and Vascular Disease: Pretrex and Diamicron Modified Release Controlled Evaluation (ADVANCE) post-trial follow-up study did not observe such beneficial effects [26]. There were many aspects that led to opposing results among these studies, and a crucial point was the difference in ages of those enrolled. The mean age of participants at enrollment was 27 years in the DCCT, 53 in the UKPDS, and 66 in the ADVANCE trial. In other words, older patients in the ADVANCE trial could not achieve any long-term benefit from intensive glucose control, likewise for younger patients in the DCCT and UKPDS studies. In addition, the original beneficial effect of intensive glucose control in the ADVANCE trial was primarily due to reductions in progression of renal disease, which is of no additional benefit to patients with existing ESRD [27, 28]. Though there is no randomized controlled trial, these results strongly raise doubts about strict control in older patients under dialysis. The possible reasons might include the higher risk of hypoglycemia in older ESRD patients. Additionally, for older patients whose life expectancy is relatively short and who have higher competing risks, there would be less benefit from reduction of micro- and macro-vascular complications that require longer periods before improvements can be observed. Furthermore, older patients with lower HbA1c levels may suffer from poor nutritional status, frailty, or sarcopenia, each of which may contribute to an elevated mortality risk [29].

On the other hand, our results suggest that glycemic control measured by HbA1c levels might have different importance in HD versus PD patients. The glycemic control significantly affected mortality up to the later ages up to 65 years old, among PD patients. The deaths from infection were predominant in the PD patients, and patients with HbA1c≥8% showed higher mortality due to infection. These results suggest that poor glycemic control negatively affects survival in PD patients because of higher risk of infection. A prospective observational study investigating diabetic PD patients showed similar results; non-cardiovascular deaths mainly caused by infection were most frequent in patients with the highest tertile of HbA1c [17]. The authors suggested that diabetic PD patients might be more vulnerable to infection due to the hypertonic glucose solution used for PD, production of advanced glycation end-products and elimination of phagocytes and immunoglobulins during frequent exchanges of PD fluid. Apart from infection risk, glycemic control is also important for the preservation of residual renal function (RRF) in the PD population. Compared to the HD population, patients undergoing PD have greater chances of preserving of RRF which is known to be important for better overall survival [30]. Based on our data and previous evidence, we suggest that adequate glucose control of at least HbA1c<8% should be considered in PD patients under 65 years of age.

This analysis included a large number of patients in a prospective cohort. Our results are significant because this is the second report providing evidence that glycemic control varies according to age in diabetic patients under dialysis. Our data suggest, for the first time, that glycemic control is important in the PD population until later periods of life compared to HD patients.

Certain limitations should be considered in the interpretation of our findings. First, the usefulness of HbA1c values in the dialysis population is currently under debate. Due to the reduced life span of erythrocytes and exogenous erythropoietin, there may be less time for hemoglobin glycosylation to occur in ESRD patients. Nevertheless, HbA1c is easily measured and currently used in clinical practice worldwide. We believe it is worthwhile to have evidence available for setting standards for HbA1c levels in the dialysis population. Second, we performed the analyses using HbA1c levels that were checked only once in each patient. It might not represent long-term glycemic control of the patients. In addition, HbA1c was measured at each center without using a uniformed method. Lastly, we suggested target HbA1c level solely based on patient mortality not considering other important complications of diabetes such as retinopathy and neuropathy which enormously affect patient quality of life. To set a goal HbA1c for dialysis population, further studies are needed.

In summary, glycemic control is important in the diabetic ESRD patient, although it might have different effects according to the type of dialysis and age. In patients older than 65 years, glycemic control was not associated with mortality. Considering both age and type of dialysis, glycemic control significantly affected mortality in patients under 55 years old in the HD group, while it was significant under 65 years old in the PD group. In the absence of trials, and limitations notwithstanding, our results support that glycemic control should be emphasized in the PD population for later periods of their life compared patients on HD. Above all, risks of hypoglycemia and benefits of glycemic control must be balanced and HbA1c goals individualized in all patients in real-life clinical practice.

## Supporting Information

S1 Fig Kaplan-Meier survival curves for all-cause mortality by HbA1c (%) (A) Patients with dialysis duration less than 1 year, (B) 1–3 years, (C) 3-6years, and (D) more than 6 years. (PDF)

S2 Fig Kaplan-Meier survival curves for all-cause mortality by change of HbA1c (%) from baseline to 1 year follow-up (A) All patients whose follow-up data were available (N = 574), (B) Incident patients (N = 270), and (C) Prevalent patients (N = 304). (PDF)

S1 Text Thirty-one centers participating in Clinical Research Center for End Stage Renal Disease (in alphabetical order). (PDF)
