# Supplementary material for: Glycemic Control and Mortality in Diabetic Patients Undergoing Dialysis Focusing on the Effects of Age and Dialysis Type: A Prospective Cohort Study in Korea
Source: PLoS One. 2015 Aug 18;10(8):e0136085. doi: 10.1371/journal.pone.0136085 (PMC4540490; doi:10.1371/journal.pone.0136085)
Supplement: S1 Fig — (PDF) [file pone.0136085.s001.pdf]

S1 Figure. Kaplan-Meier survival curves for all-cause mortality by HbA1c (%) (A) Patients with dialysis duration less than 1 year, (B) 1-3 years, (C) 3-6 years, and (D) more than 6 years

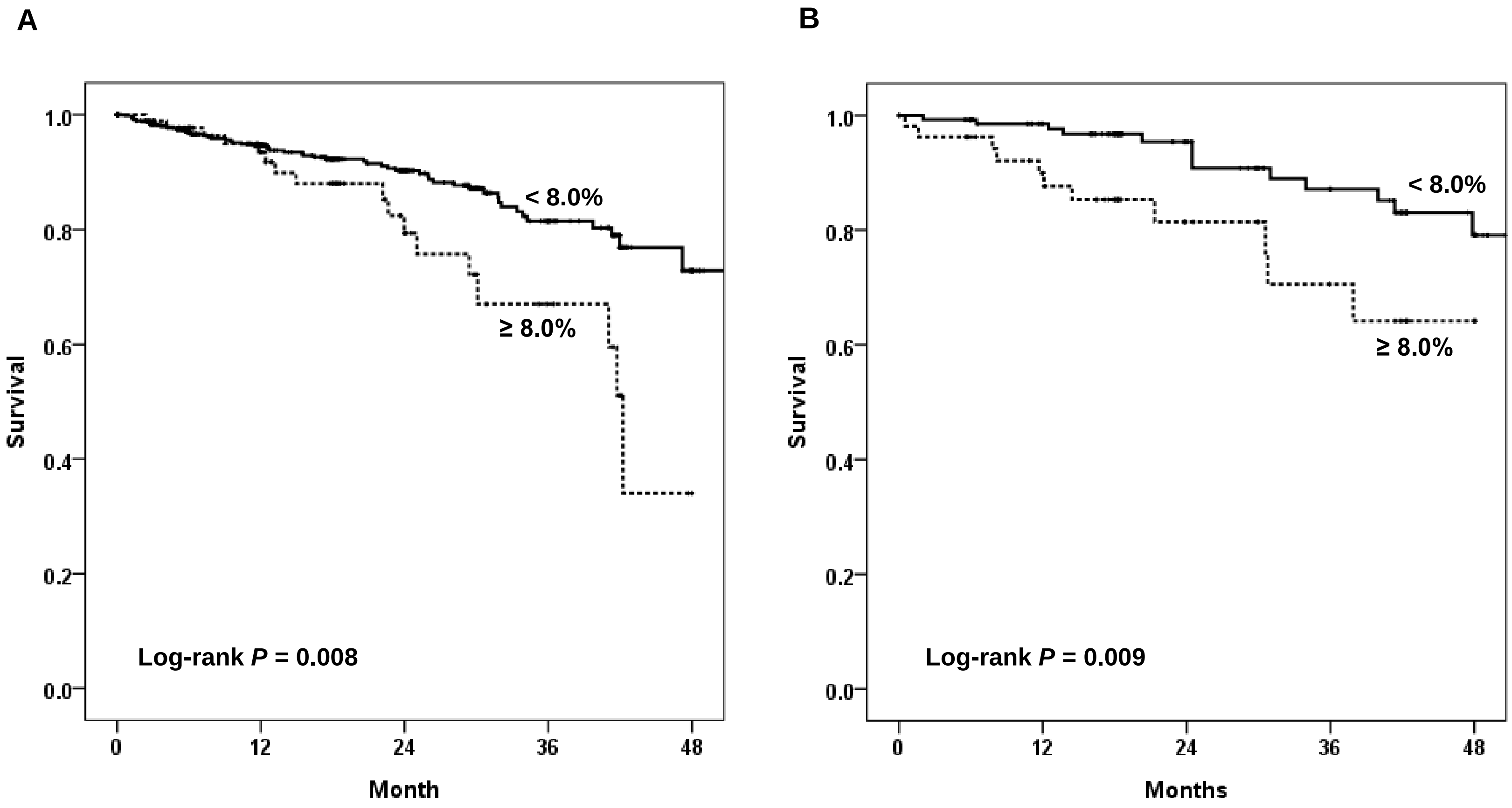

C

D

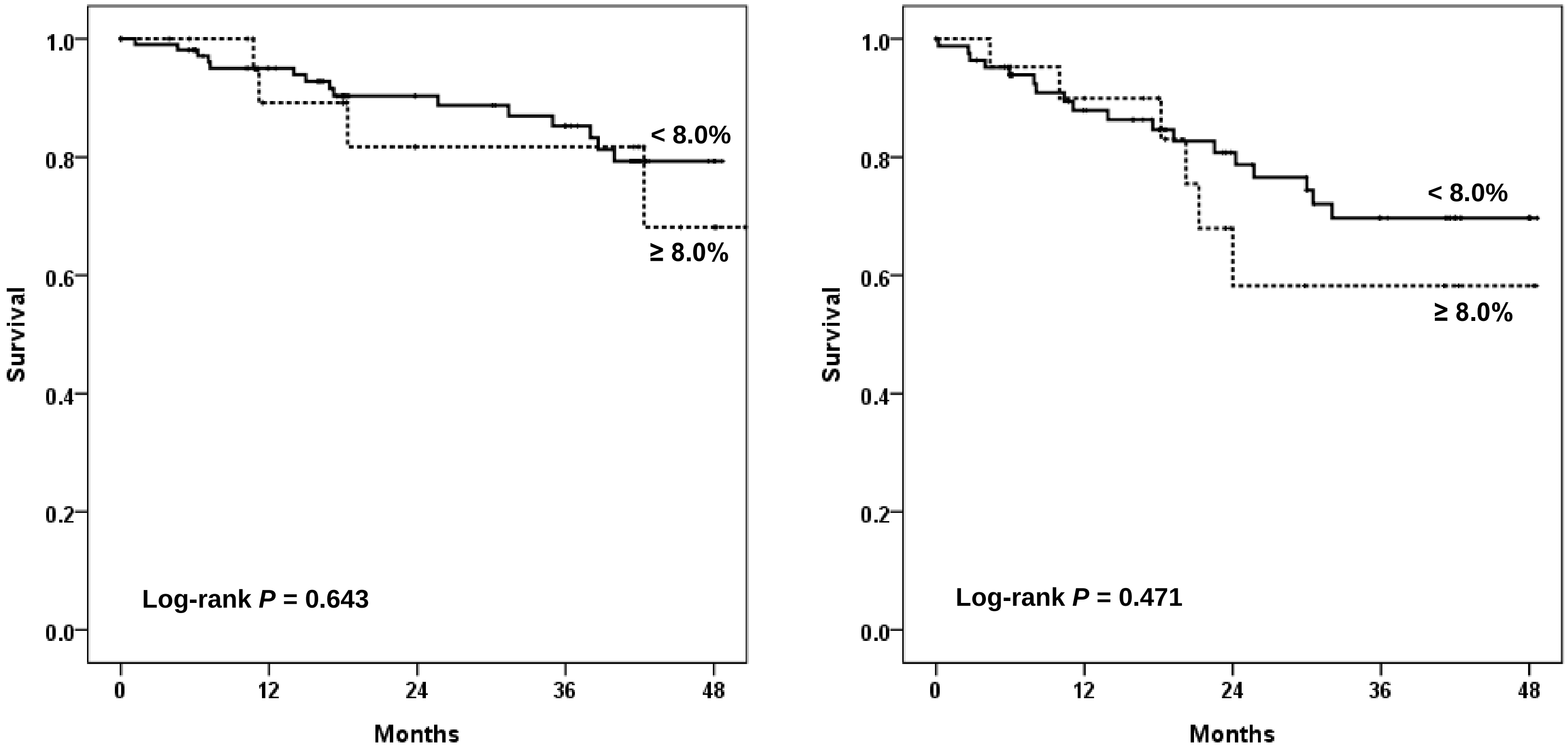
1.0
0.8
0.6
0.4
0.2
0.0
Survival
< 8.0%
≥ 8.0%
Log-rank P = 0.643
0
12
24
36
48
Months
1.0
0.8
0.6
0.4
0.2
0.0
Survival
< 8.0%
≥ 8.0%
Log-rank P = 0.471
0
12
24
36
48
Months
