# Supplementary material for: Glycemic Control and Mortality in Diabetic Patients Undergoing Dialysis Focusing on the Effects of Age and Dialysis Type: A Prospective Cohort Study in Korea
Source: PLoS One. 2015 Aug 18;10(8):e0136085. doi: 10.1371/journal.pone.0136085 (PMC4540490; doi:10.1371/journal.pone.0136085)
Supplement: S2 Fig — (PDF) [file pone.0136085.s002.pdf]

S2 Figure. Kaplan-Meier survival curves for all-cause mortality by change of HbA1c (%) from baseline to 1 year follow-up (A) All patients whose follow-up data were available (N = 574), (B) Incident patients (N = 270), and (C) Prevalent patients (N = 304)

A

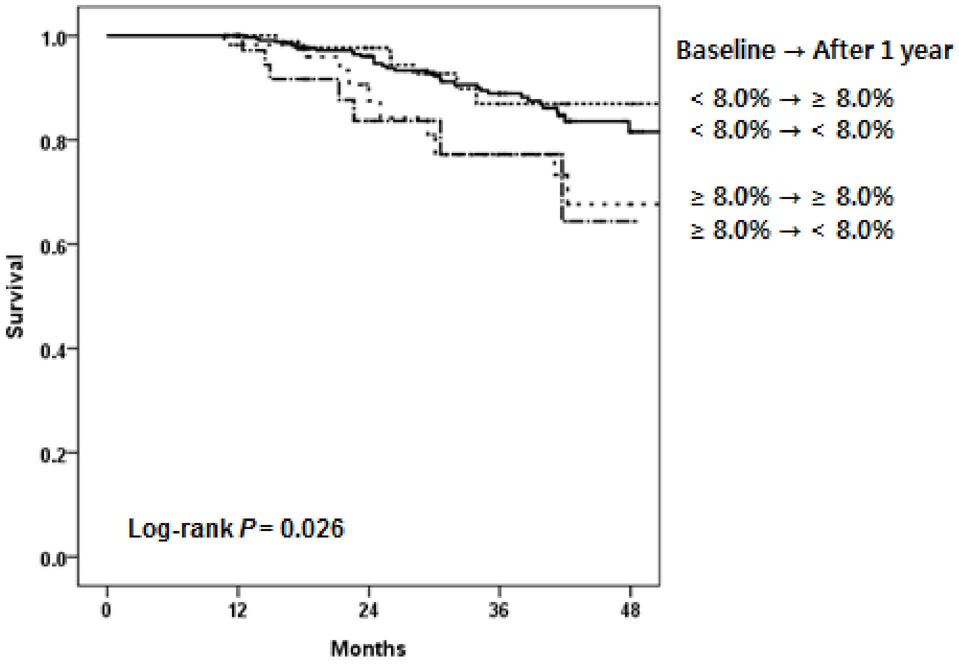

B

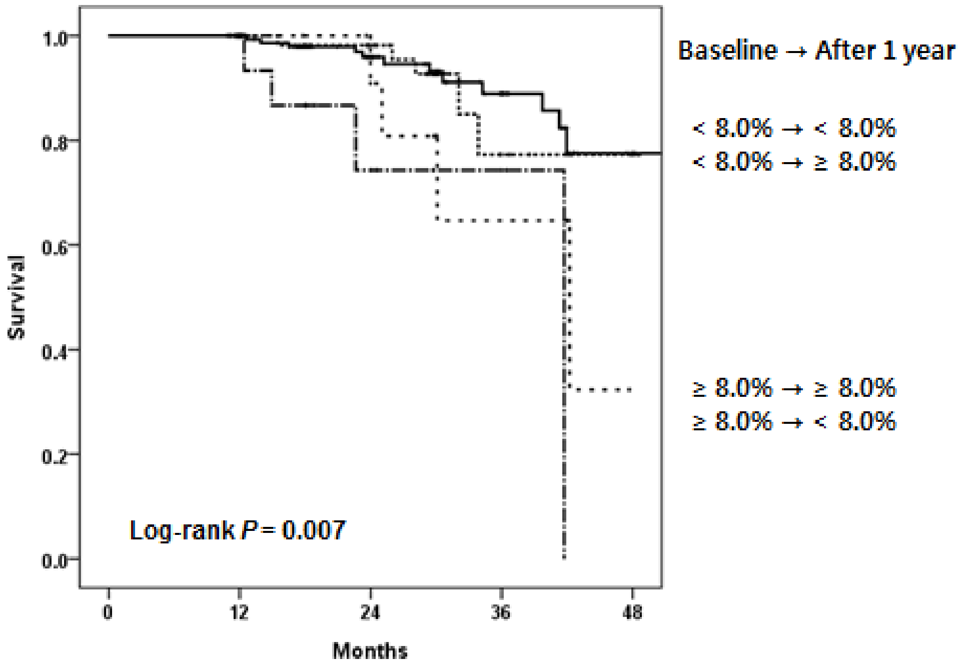

C

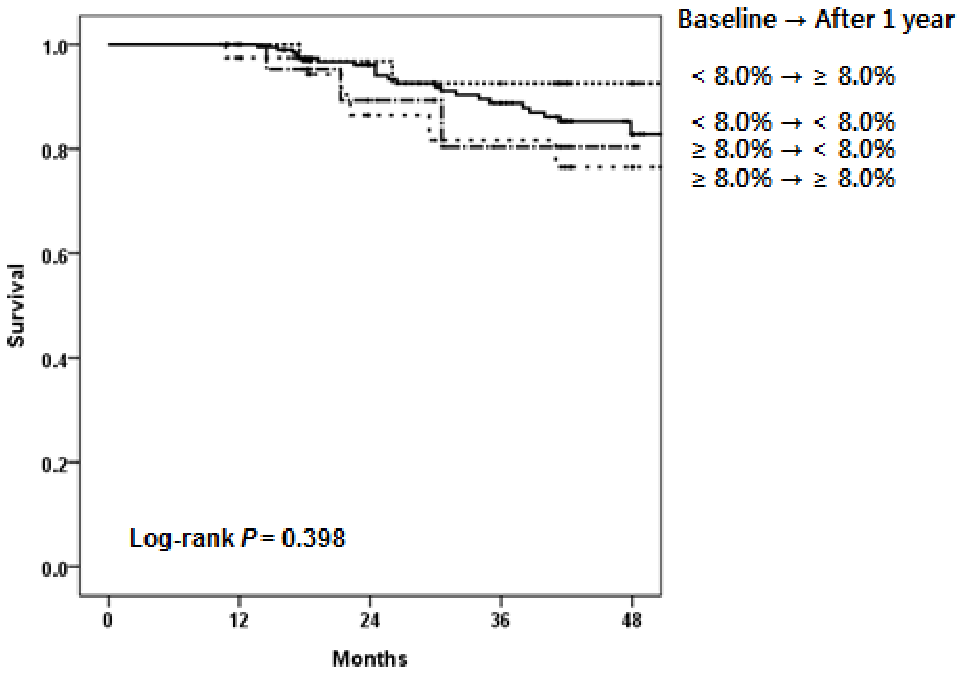
Survival
1.0
0.8
0.6
0.4
0.2
0.0
0
12
24
36
48
Months
Log-rank P = 0.398
Baseline → After 1 year
< 8.0% → ≥ 8.0%
< 8.0% → < 8.0%
≥ 8.0% → < 8.0%
≥ 8.0% → ≥ 8.0%
