# Supplementary material for: Glycemic Control and Mortality in Diabetic Patients Undergoing Dialysis Focusing on the Effects of Age and Dialysis Type: A Prospective Cohort Study in Korea
Source: PLoS One. 2015 Aug 18;10(8):e0136085. doi: 10.1371/journal.pone.0136085 (PMC4540490; doi:10.1371/journal.pone.0136085)
Supplement: S1 Text — (PDF) [file pone.0136085.s003.pdf]

S1 Text. Thirty-one centers participating in Clinical Research Center for End Stage Renal Disease (in alphabetical order)

The Catholic University of Korea, Bucheon St. Mary's Hospital; The Catholic University of Korea, Incheon St. Mary's Hospital; The Catholic University of Korea, Seoul St. Mary's Hospital; The Catholic University of Korea, St. Mary's Hospital; The Catholic University of Korea, St. Vincent's Hospital; The Catholic University of Korea, Uijeongbu St. Mary's Hospital; Cheju Halla General Hospital; Chonbuk National University Hospital; Chonnam National University Hospital; Chung-Ang University Medical Center; Chungbuk National University Hospital; Chungnam National University Hospital; Dong-A University Medical Center; Ewha Womans University Medical Center; Fatima Hospital, Daegu; Gachon University Gil Medical Center; Inje University Pusan Paik Hospital; Kyungpook National University Hospital; Kwandong University College of Medicine, Myongji Hospital; National Health Insurance Corporation Ilsan Hospital; National Medical Center; Pusan National University Hospital; Samsung Medical Center, Seoul; Seoul National University, Boramae Medical Center; Seoul National University Hospital; Seoul National University, Bundang Hospital; Yeungnam University Medical Center; Yonsei University, Severance Hospital; Yonsei University, Gangnam Severance Hospital; Ulsan University Hospital; Wonju Christian Hospital
